# Facile and Electrically Reliable Electroplated Gold Contacts to p-Type InAsSb Bulk-Like Epilayers

**DOI:** 10.3390/s21165272

**Published:** 2021-08-04

**Authors:** Sebastian Złotnik, Jarosław Wróbel, Jacek Boguski, Małgorzata Nyga, Marek Andrzej Kojdecki, Jerzy Wróbel

**Affiliations:** 1 Institute of Applied Physics, Military University of Technology, 2 Kaliskiego Str., 00-908 Warsaw, Poland; jaroslaw.wrobel@wat.edu.pl (J.W.); jacek.boguski@wat.edu.pl (J.B.); malgorzata.nyga@wat.edu.pl (M.N.); wrobel@ifpan.edu.pl (J.W.); 2 Institute of Mathematics and Cryptology, Military University of Technology, 2 Kaliskiego Str., 00-908 Warsaw, Poland; marek.kojdecki@wat.edu.pl; 3 Institute of Physics, Polish Academy of Sciences, Aleja Lotników 32/46, 02-668 Warsaw, Poland

**Keywords:** InAsSb, gold electrodeposition, TLM, specific contact resistivity, Hall effect measurement

## Abstract

Narrow band-gap semiconductors, namely ternary InAsSb alloys, find substantial technological importance for mid-infrared application as photodetectors in medical diagnostics or environmental monitoring. Thus, it is crucial to develop electrical contacts for these materials because they are the fundamental blocks of all semiconductor devices. This study demonstrates that electroplated gold contacts can be considered as a simple and reliable metallization technology for the electrical-response examination of a test structure. Unalloyed electroplated Au contacts to InAsSb exhibit specific contact resistivity even lower than vacuum-deposited standard Ti–Au. Moreover, temperature-dependent transport properties, such as Hall carrier concentration and mobility, show similar trends, with a minor shift in the transition temperature. It can be associated with a difference in metallization technology, mainly the presence of a Ti interlayer in vacuum-deposited contacts. Such a transition may give insight into not only the gentle balance changes between conductivity channels but also an impression of changing the dominance of carrier type from *p*- to *n*-type. The magnetotransport experiments assisted with mobility spectrum analysis clearly show that such an interpretation is incorrect. InAsSb layers are strongly *p*-type dominant, with a clear contribution from valence band carriers observed at the whole analyzed temperature range. Furthermore, the presence of thermally activated band electrons is detected at temperatures higher than 220 K.

## 1. Introduction

Carrier injection from an electrode to a semiconductor medium has routinely been a central issue in any common optoelectronic device. Metallization acting as electrical contacts to narrow band-gap (NBG) semiconductors is an integral part of devices based on such materials and determines device performances and their reliability. Among the A^III^–B^V^ NBG semiconductors are arsenides (e.g., InAs and GaAs) and antimonides (e.g., InSb and GaSb) as well as their ternary alloys (e.g., InAs_1−x_Sb_x_), considered as crucial materials for mid-infrared (MIR) optoelectronic devices, operating with a radiation of wavelength between 2 and 14 µm [1]. The substitution of antimony sites in InSb with isovalent arsenic reduces the energy gap of an InAsSb solid solution to a value lower than the energy gap of their constituent binary compounds, being the ternary alloy with the lowest energy gap among the A^III^–B^V^ semiconductors [2]. The development of InAsSb alloys led to replacing HgCdTe in MIR applications due to superior bond strengths, material stability, doping capability or high-quality A^III^–B^V^ substrates [2]. The MIR photodetectors find considerable technological importance in medical diagnostics, IR imaging, environmental monitoring and chemical sensing [3,4,5,6]. Recently, InAsSb-based detector technology has been extended to the development of photodiodes with a variety of configurations such as *n*-i-*p* structure [3,4] or to the XBn barrier structures [7].

Generally, an InAsSb-based bulk photodiode grown epitaxially is composed of certain layers with a particular conductivity type, while usually a top *p*-type cladding is used as a contact layer [8]. Therefore, in device processing, it is crucial to obtain ohmic contacts at the metal/semiconductor interface, being a fundamental building block of any semiconductor device. This metal/semiconductor tandem is frequently called a Schottky barrier device with defined current–voltage (I–V) characteristics [9,10]. Contact resistance, in particular specific contact resistivity (*ρ_c_*), is an important parameter characterizing metal/semiconductor interfaces and metal contacts, a practical quantity describing real contact.

Regarding metallization studies on InAsSb, either bulk-like or superlattice device structures, mostly subsequentially deposited Ti, Pt and Au films were used (in some cases, without a Pt interlayer) [5,11,12,13,14]. In such multi-layered metallization, the Ti film acts as an adhesive and a barrier component but Pt prevents Au penetration into the underlying layers [15]. The thermal stability studies conducted by Lee et al. [16] on InAs/graded InGaAs structure revealed that the In atoms diffuse and penetrate into the 110 nm thick Ti layer, leading to InAs film decomposition at 350 °C. Other more complex Au-containing metallization schemes were tested as well on InAs-based structures using distinct adhesive/barrier layers such as Pd, Co, Ni, etc. [17,18,19]. Nevertheless, Au is normally the main constituent of metallization schemes due to high electrical conductivity and excellent corrosion resistance [20] and is vital in processing semiconductor devices.

There are several methods of metal deposition, and mostly vacuum technologies (physical vapor deposition) have been implemented, namely thermal evaporation or sputtering. These dry coating methods are considered beneficial to wet bath technologies due to environmental issues. However, the well-established electrochemical deposition by electrolytic processes is an attractive method for the integration of metals with semiconductors [21]. The deposition of a metallic coating by these processes, either in an aqueous or non-aqueous electrolytic environment, is a valuable alternative to commonly used dry techniques under vacuum conditions [22], mainly because of lower system costs and process time. It can be particularly important for the electrical characterization of device test structure.

Recently, Au-coated surfaces garnered increasing interest in novel applications, such as fuel cells, electrochemical sensing, energy storage and catalysis [23]. Electrochemically deposited Au nanoparticles appear to be of high importance due to high catalytic activity in chemical reactions, synthesized and incorporated into distinct surfaces [24,25,26,27,28]. The electrodeposition of Au also provides a good platform for membrane technologies suitable for gas-sensing processes [29]. Therefore, electrochemistry has been demonstrated as one of the most accessible, versatile and cost-effective approaches for nanostructuring semiconductors in a controlled manner. Moreover, Au electroplating has been developed for centuries, having an enormous impact on the electronic industry [30].

The current work presents a comparative study of electroplated and vacuum-deposited Au contacts to *p*-type ternary InAsSb bulk-like device structures for complex electrical characterization, namely Hall effect measurements. Knowledge about transport properties, in particular the precise description of electrical carrier characteristics, is important for the design of complex structures meant for applications. Prior to actual temperature-dependent magnetotransport measurements, the contact resistance measurement technique (also called Shockley method [31]) was implemented to evaluate *ρ_c_*. For this purpose, I–V characteristics were collected in a broad temperature range of 10–300 K using a multiple-contact test structure. The temperature-dependent Hall effect and resistivity measurements were conducted at a constant magnetic field of 0.54 T as well as at selected temperatures with a magnetic field scanning up to 10.5 T for mobility spectrum analysis. Such a measurement approach assisted with transport properties, and interpretation leads to a more reliable comparison of metallization technologies.

## 2. Materials and Methods

### 2.1. p-InAsSb Epilayer Growth and Processing

**Figure 1:**
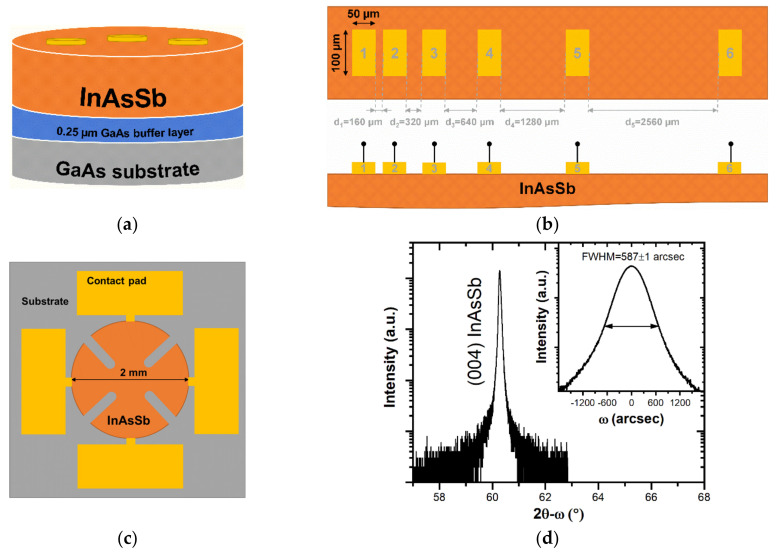
(**a**) Scheme presenting the architecture of the device structure under study. (**b**) Top-view and lateral (cross section) sketch of a linear TLM test structure with marked dimensions. (**c**) A cloverleaf van der Pauw geometry scheme with marked measurement area, contact pads and substrate. (**d**) (004) X-ray scans in ω-2θ and ω directions of InAsSb.

The approximately 5.2 µm-thick Be-doped InAsSb layer with InAs_0.81_Sb_0.19_ composition was grown by RIBER Compact 21-DZ solid-source molecular beam epitaxy (MBE) system on a 2-inch semi-insulating GaAs substrate. The specification of this particular MBE system is described in more detail elsewhere [32,33]. After the degassing process of GaAs substrate (thermal desorption of oxides), a 250 nm-thick GaAs buffer layer was initially grown at a 670 °C manipulator temperature with 0.9 µm/h growth rate to improve the substrate surface quality. The actual Be-doped InAsSb layer was grown at 425 °C with 0.5 µm/h growth rate and Be cell set at 850 °C. The other growth conditions were listed in our previous work [34]. Figure 1a depicts the scheme, presenting the architecture of the device structure under study.

The *ρ_c_* and sheet resistance (*R_SH_*) were determined using a linear transmission line model (TLM) method. This method is commonly used in devices for metal/semiconductor contacts examination involving I–V measurements on the adjacent contacts with variable spacing between them [9,31]. The TLM test structures were fully processed by including positive photolithography and contact deposition on 4 × 6 mm samples. Each sample consisted of six rectangular contacts with length (L) equal to 100 µm and width (Z) equal to 50 µm, separated by distinct distances (d_1–5_) ranging from 160 to 2560 µm (see schematic presented in Figure 1b). Au-containing contact deposition was conducted by the two methods described in Section 3.1. Regardless of the method, short wet etching was performed to remove the native oxide on the surface. A detailed description of the sample’s preparation and metallization was presented in our previous work [34].

The Hall effect measurements were carried out on the highly symmetrical samples in the cloverleaf van der Pauw geometry, with the maximum resistance value in four-point terminals of 0.12%. In such a test structure, the active area in the center is connected by four pathways to four connection pads around its perimeter (see Figure 1c). The four-terminal test structures were defined lithographically. The remaining surface of the sample was etched to the GaAs substrate in a mixture of orthophosphoric acid, citric acid, hydrogen peroxide and water solution [35]. Each contact area was bonded with a 25-µm-diameter Au wire using a wire bonder machine and then attached to the sample holder pins.

### 2.2. Characterization Methods

A high-resolution X-ray diffractometer (XRD) with Cu Kα_1_ radiation, PANalytical X’Pert MRD, was used to evaluate the structural characteristics of the structure under study. The ω-2θ and ω scans were collected.

The electrical characterization was conducted by a superconducting 16 T Cryogen-Free Magnet System (CFMS) equipped with cryostat, fabricated by Cryogenic Ltd. (London, UK). The samples fixed to holders and located on a variable temperature insert were directly placed in the circulating high-purity He gas (coolant agent) at a constant pressure (closed cycle mode). Such conditions ensure temperature stabilization (≤50 mK for 5–300 K), monitored by Cernox^TM^ sensor (Lake Shore Cryotronics, Inc., Westerville, OH, USA).

The I–V characteristics and differential resistance (*R_d_*; defined as derivative of a voltage with respect to a current) were measured using Source Measure Unit model Agilent B2902A two-channel programmable multimeter. The measurements were conducted in the temperature range of 10–300 K. The sample was biased by a voltage source with simultaneous current measurement to collect required data during the linearly changed temperature at ramp rate of 0.5 K/min. This approach ensures acquisition of at least fifteen I–V characteristics for each contact pair to average for a certain temperature (every 5 K; ±2 K).

The Hall effect measurements were performed in the temperature range of 10–300 K (ramp rate of 0.25 K/min) at a constant magnetic field (*B*) of 0.54 T as well as at variable *B* up to 10.5 T. The current source was a Keithley 2400 Source Metter, whereas voltages were gathered by a Keithley 2182A nanovoltmeter. The Hall effect sample structure was placed in the center of the ±16 T electromagnet solenoid, where the *B* homogeneity is greater than ≤0.1% total variation over a 10-mm-diameter sphere. The van der Pauw test structures were electrically pre-examined in the temperature range of 10–300 K on cooling to assess the symmetry and linearity of I–V characteristics of all contact pairs and to select appropriate bias conditions for the resistivity and Hall effect measurements [36].

The mobility spectrum analysis (MSA) framework was used to identify different conductivity channels, which are responsible for electronic transport in bulk and layered materials. The so-called mobility spectrum, *S(μ)* ≥ 0, is calculated from magnetic-field-dependent Hall effect measurements at 220, 283 and 300 K [37]. It is assumed that conductivity tensor components (*σ_xx_* and *σ_xy_*) can be expressed as integrals of the Drude-like terms:

(1) $$\sigma_{xx}\left(B\right)=\int_{-\infty }^{\infty }\frac{S\left(\mu \right)\;}{1+\mu^{2}B^{2}}\;d\mu ,$$

(2) $$\sigma_{xy}\left(B\right)=\int_{-\infty }^{\infty }\frac{S\left(\mu \right)\;\mu B}{1+\mu^{2}B^{2}}d\mu .$$

where *μ* stands for a mobility of carriers. The shape of continuous function *S(μ)* provides a deeper insight into the transport mechanisms present in the conducing sample and delivers more information as compared to the low-field data alone. Usually, separate spectral peaks are interpreted as distinct conduction channels related to electrons (*μ* < 0) or holes (*μ* > 0), including surface conductivity, impurity bands or interface transport channels in layered structures [38].

## 3. Results and Discussion

A high demand for InAsSb technology is driven by a need to further develop environmentally friendly MIR detection modules compliant with the RoHS (Restriction of Hazardous Substances) directive. Historically, the InAs_0.2_Sb_0.8_-based photoconductor with a cut-off wavelength of 3.65 µm was first demonstrated at the end of the 1980s, showing the feasibility of InAsSb integration with Si technology [39]. Recently, gallium-free InAs/InAsSb superlattice photodetectors for MIR photonics were demonstrated with a cut-off wavelength of about 5 µm [5,40]. Generally, photodetectors composed of InAsSb are under constant expansion on a market, requiring device processing for electrical characterization with a rather simple and fast metallization methodology.

In this work, prior to metallization, the InAsSb epilayer under study was initially evaluated by XRD (see Figure 1d). The presented symmetrical (004) diffraction line of InAsSb is sharp, proving the abruptness of InAsSb interface with underlayers. Additionally, the measured rocking curve in ω direction (shown as inset) is found to be approximately 587 arcsec, confirming a relatively good crystalline quality of as-grown InAsSb.

### 3.1. Metallization Approaches

**Figure 2:**
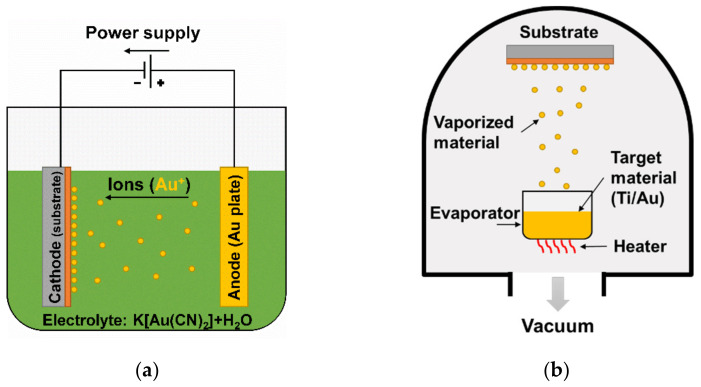
Schematic diagrams of two metallization methods used in the present work: (**a**) wet technology (Au electrodeposition) and (**b**) dry technology (Ti–Au vacuum evaporation).

The Au contact deposition was conducted by two methods: (i) wet technology, namely electroplating (Figure 2a), and (ii) dry technology, namely vacuum evaporation (Figure 2b). In the first one, an approximately 600 nm thick Au contact layer was obtained using potassium dicyanoaurate, K[Au(CN)_2_], and a water solution as an electrolyte under 350–375 µA/cm^2^ current density. K[Au(CN)_2_] solution was utilized for the deposition of Au on *p*- and *n*-type GaAs nearly three decades ago [41,42,43]. The same protocol was used in our previous work on binary arsenide, namely InAs [34], proving that these contacts adhere well to arsenide epilayers.

Regarding Au electrodeposition on A^III^–As, there have been only a few reports, not necessarily on InAsSb but rather on GaAs [41,42,43]. A study on the electrochemical behavior of distinct GaAs surfaces in an Au-containing aqueous electrolyte solution revealed that the nucleation mechanism of Au on these surfaces depends on the chemical composition of the surface [22,44]. It was revealed that a smooth, mirror-like Au layer with good adhesion to the GaAs surface can be obtained. Moreover, it was stated that, in the nucleation of Au on (100) n-GaAs, both Ga and As atoms act as nucleation centers.

The second approach, vacuum evaporation, is a standard dry method for semiconductor device metallization. Here, a 5-nm-thick adhesive Ti (99.995%) interlayer prior to a 150-nm-thick Au (99.999%) contact layer were coated on InAsSb at approximately 10^−6^ mbar using an Angstrom Engineering Nexdep thin film deposition system (Angstrom Engineering Inc., Kitchener, ON, Canada). The adhesive Ti layer with noble metal overlayer has been used as a bilayer for decades because Ti is known to be more chemically reactive than Au and thereby increases the adhesion as it chemically binds to the dielectric or semiconductor substrate [45]. This approach was implemented as a comparison to the Au electrodeposition.

### 3.2. Contact Resistance

**Figure 3:**
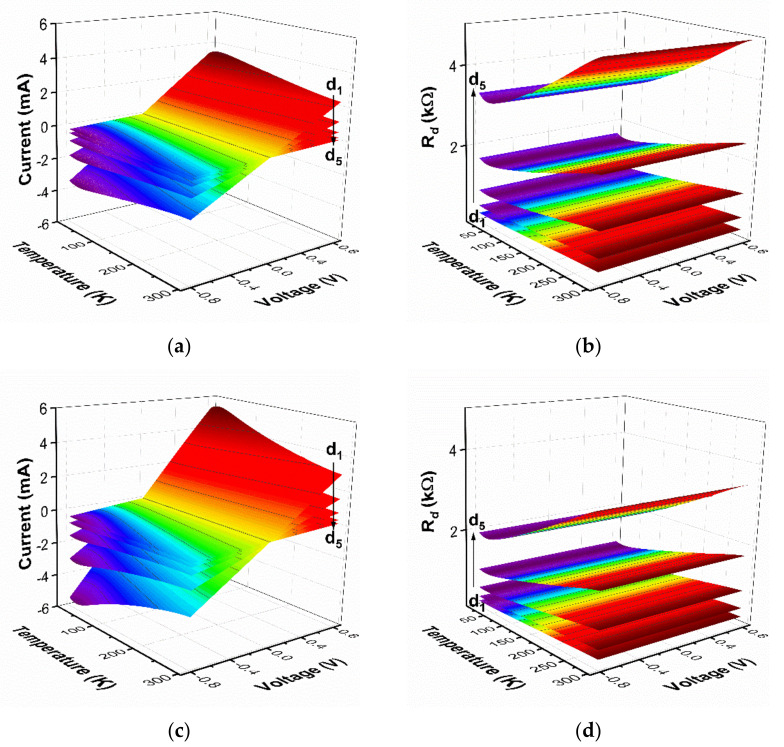
Temperature dependence of I–V and *R_d_*–V characteristics in the temperature range of 10–300 K and applied voltage of ±0.8 V for both types of metallization: (**a**,**b**) Au electroplating and (**c**,**d**) Ti-Au vacuum deposition. Curves present data collected from six rectangular contacts separated by distinct distances, where d_1_ = 160 µm, d_2_ = 320 µm, d_3_ = 640 µm, d_4_ = 1280 µm and d_5_ = 2560 µm.

*ρ_c_* is a useful parameter, a figure of merit for ohmic contacts, defining contact resistivity because it is independent of the contact area and is convenient while comparing contacts of various sizes. A multiple-contact two-terminal measurement technique with the lateral structure (TLM) was implemented to determine *ρ_c_*. The I(V,T) and *R_d_*(V,T) characteristics acquired for each pair of contacts are depicted in Figure 3a–d for the Au electroplated and Ti–Au vacuum deposited, respectively; the same axis scaling was used for comparison. The presented 3D surface-type plots show a set of data where linear I(V) curves prove no or minor Schottky contribution independently of temperature, a characteristic of the resistive element. The calculated temperature-dependent *R_d_*(V) data confirm the ohmic behavior of both types of metallic contacts, being a straight line independent of the voltage.

**Figure 4:**
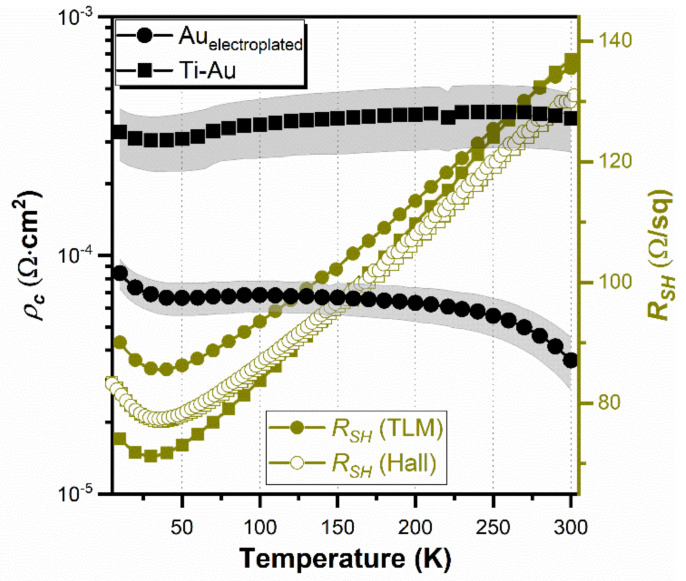
The temperature-dependent *ρ_c_* and *R_SH_* of electroplated Au and vacuum-deposited Ti–Au contacts to InAsSb. *R_SH_* derived from TLM (closed symbols) and Hall effect (open symbols) measurements are included.

Furthermore, the temperature-dependent *ρ_c_* and *R_SH_* derived from the TLM measurements were estimated for both metallization types, and the results are presented in Figure 4. For electroplated Au on InAsSb, *ρ_c_* slightly decreases with rising temperatures from 8.4 × 10^−5^ Ω·cm^2^ at 10 K to 3.6 × 10^−5^ Ω·cm^2^ at 300 K, while for vacuum deposited Ti-Au on InAsSb, it is rather constant: 3.3 × 10^−4^ Ω·cm^2^ at 10 K and 3.8 × 10^−4^ Ω·cm^2^ at 300 K. In the case of *R_SH_* derived from TLM (see Figure 4, closed symbols), a trend is very similar for both metallization types, with a maximum difference of approximately 17% at temperatures lower than 50 K. Moreover, *R_SH_* obtained from the Hall effect measurements are included in Figure 4 (open symbols), exhibiting nearly alike temperature-dependent values. Nevertheless, it can be concluded that electroplated Au on InAsSb exhibits nearly one order of magnitude lower *ρ_c_* compared to vacuum-deposited Ti–Au on InAsSb (at 300 K). A similar phenomena was already observed for *p*-type Be-doped binary InAs epilayers for certain doping level, >10^17^ cm^−3^, but to a lower extent [34]. It could be associated with a difference in the composition of epilayers under study, with ternary InAsSb being approximately 20 mol% of Sb. It was proven that Au is, in fact, not totally inert with respect to the A^III^–B^V^ semiconductor materials but rather interacts to form a variety of intermetallic compounds [46]. A recent study on Au interaction with In-based semiconductors revealed that the (001) surface of InAs is more stable than InSb towards Au-induced etching [47]. Moreover, the Au diffusion into the bulk lattice of InSb crystal is very efficient because of the lowest binding energy among studied In-based and Ga-based compounds.

The contact resistance values for metallization schemes in Sb-containing InAs-based semiconductors have been limitedly reported. For instance, Mohney and co-workers presented an extended study on distinct three- and four-layered ohmic contact metallization with Au cap layer to maintain a low metal sheet resistance to highly Be-doped InAs (>10^19^ cm^−3^) with *ρ_c_* at a level of 10^−6^ Ω·cm^2^ [17,18]. Guo et al. conducted an analysis on highly Be-doped InAs/InAsSb cap structure, yielding an ultralow ohmic contact with *ρ_c_* of 1.3 × 10^−8^ Ω·cm^2^ [19]. However, these works presented studies on alloyed contacts with complex metallization schemes and *ρ_c_* estimated solely at room temperature.

### 3.3. Magnetotransport Measurements

**Figure 5:**
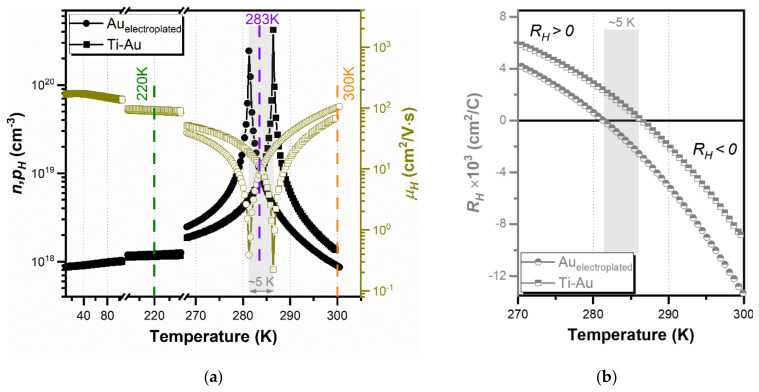
(**a**) Hall effect measurement results showing the temperature dependence of *n,p_H_* and *µ_H_* of both metallization types: electroplated Au and vacuum-deposited Ti–Au. The region of approximately 5 K difference in transition temperature between both metallization types is marked in grey. Additionally, magnetic-field-dependent measurements conducted at 220, 283 and 300 K are indicated. (**b**) *R_H_* as a function of temperature for both types of metallization, limited to the temperature range of 270–300 K. The change of *R_H_* sign is observed at 281 K for electroplated Au and 286 K for Ti–Au.

The Hall effect and resistivity measurements are important techniques to directly obtain the total charge carrier concentration (*n,p_H_*), averaged Hall mobility (*µ_H_*) as well as *R_SH_* (see Figure 4; open symbols) of a particular specimen. These measurements are routinely used for electronic materials at room temperature or in a temperature-dependent measurement mode, typically at low magnetic fields, <1 T. Figure 5a shows variations in the absolute values of *n,p_H_* and *µ_H_* as a function of temperature ranging from 10 to 300 K for both metallization types. The *n,p_H_* and *µ_H_* for Au-electroplated InAsSb are ~9 × 10^17^ cm^−3^ and 102 cm^2^/V·s at 300 K and ~1.2 × 10^18^ cm^−3^ and 88 cm^2^/V·s at 220 K. In the case of vacuum-deposited Ti–Au to InAsSb, these parameters are ~1.4 × 10^18^ cm^−3^ and 68 cm^2^/V·s at 300 K and ~1.1 × 10^18^ cm^−3^ and 95 cm^2^/V·s at 220 K. It can be clearly observed that InAsSb exhibits similar trends in temperature-dependent transport characteristics, independently of metallization. However, Hall data exhibit distinct conductions, where transport is governed by holes at lower temperatures, <280 K, and as temperature increases near room temperature, the transport is dominated by electrons. This anomalous behavior was already observed in the 1990s [48,49]. It was later stated that the large mobility of electrons compared to holes results in a dominant contribution of electrons to the Hall voltage, though electrons are minority carriers in the bulk [50]. It is reflected in a change in the sign of the Hall coefficient (*R_H_*); see Figure 5b. This low-field parameter can be used to obtain the charge concentration and mobility of current carriers only for a single type of electronic transport, which is not the case for our structures, intentionally *p*-type doped InAsSb. Moreover, there is an approximately 5 K shift in the transition temperature: 281 K for electroplated Au and 286 K for Ti–Au. It can be associated with a difference in metallization technology, mainly the presence of a Ti interlayer in vacuum-deposited contacts. Consequently, it slightly changes the Fermi level position, reflected in a transition temperature shift. The calculated band profiles for *p*-type InAs_0.81_Sb_0.19_ (band-gap energy of 0.276 eV) can be found elsewhere [51]; this particular InAsSb composition was used as an absorber in heterostructure devices operating up to a 5.3 µm cut-off wavelength at 230 K. Thus, we extended our comparisons by collecting high *B* data and by applying MSA.

**Figure 6:**
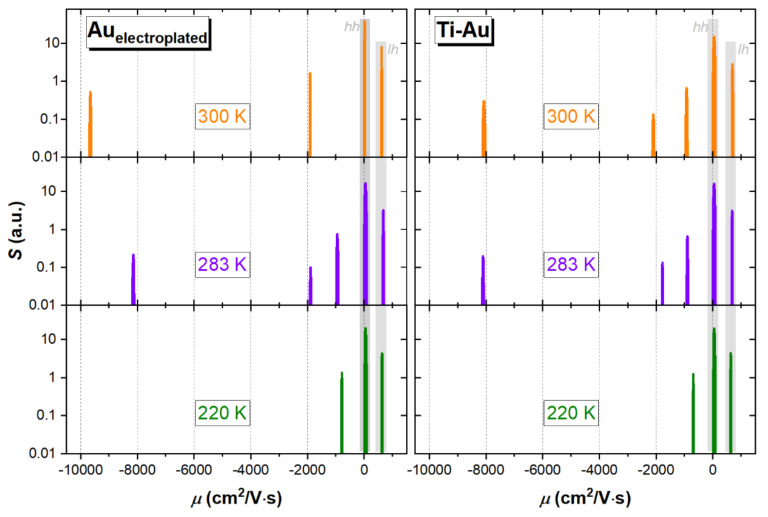
Carrier mobility spectra, *S(µ)*, of InAsSb with both types of metallization, collected at 220, 283 and 300 K.

Interpretation of the above results is not straightforward because not all carriers have the same mobility. In modern materials containing multiple carrier mobilities, an analysis of magnetic-field-dependent Hall measurements is needed. To obtain mobility spectrum, which is not an easy task, several numerical approaches exist in the literature [37,52]. In this work, we applied an original MSA method, for which we adopted constrained optimization algorithms, available in the SciPy module of a Python ecosystem [53]. The specifics of our approach, implemented here, will be presented in separate paper. The calculated mobility spectra, *S(µ)*, for InAsSb structures with both types of metallization are depicted in Figure 6 acquired at three distinct temperatures: 220, 283 and 300 K. The presented *S(µ)* are normalized to zero-field conductivity and are based on magnetoconductivity data collected in a high magnetic field up to *B_max_* = 10.5 T.

As expected, InAsSb layers are strongly *p*-type dominant with hole mobility *μ_hh_* = 45 cm^2^/V·s and concentration *p_hh_* = 1.79 × 10^18^ cm^−3^ at T = 283 K (for the sample with electroplated Au contacts). Clearly, the contribution from valence band light holes (*μ_lh_* = 675 cm^2^/V·s, *p_lh_* = 1.80 × 10^17^ cm^−3^ at 283 K) is also observed at the whole analyzed temperature range. Furthermore, the presence of thermally activated band electrons, with mobility *μ_e_* = 8143 cm^2^/V·s and concentration *n_e_* = 1.13 × 10^14^ cm^−3^, at 283 K, is detected at higher temperatures, >220 K. Very similar parameters, which are responsible for the negative value of Hall voltage at low magnetic fields, were obtained for InAsSb with standard Ti–Au electrical contacts; see Figure 6. Nevertheless, it can be stated that electroplated Au as contact metallization does not change the properties of all conductivity channels as compared to the vacuum deposition method. Slight differences in mobilities and concentrations among both types of metallization may be related to the differences in Fermi level position within valence bands.

Interestingly, spectra obtained by MSA reveal the presence of two additional, electron-like carrier species, which may also contribute to the negative sign of low-field *R_H_* parameter. We believe that the lower mobility peak (approximately 1000 cm^2^/V·s), observed for all temperatures, is related to the warping of the InAsSb heavy-hole band. Another higher mobility peak (approximately 2000 cm^2^/V·s), which appears at ≥283 K, may indicate the presence of a thermally activated surface inversion layer or *n*-type interface conduction. This subject definitely requires further studies; however, the appearance of both additional electron-like carriers is not related to the processing of electrical contacts.

## 4. Conclusions

The presented study was intended to compare the simple Au-electroplated route and standard vacuum-deposited Ti–Au for metallization to the InAsSb epilayer. The following main conclusions can be drawn: (i) unalloyed electroplated Au ohmic contacts exhibit *ρ_c_* lower than Ti–Au, approximately one order of magnitude; (ii) the carrier transport properties of InAsSb show similar temperature-dependent trends, independently of metallization type; and (iii) InAsSb, either with electroplated Au or Ti–Au contacts, is characterized by multiple populations of distinct carrier species with strong *p*-type dominance. It is also worthy to mention that the carrier characteristics obtained from MSA are not susceptible to *R_SH_* as standard Hall effect measurements at low *B*. Ultimately, it can be concluded that electroplated Au might be considered as facile and a reliable metallization technology for InAsSb-based test structure examination.

## Data Availability

Not applicable.

## References

[1] Rogalski A., Rogalski A. (2018). III–V Detectors. Infrared and Terahertz Detectors.

[2] Rogalski A., Martyniuk P., Kopytko M., Madejczyk P., Krishna S. (2020). InAsSb-Based Infrared Photodetectors: Thirty Years Later On. Sensors.

[3] Tetyorkin V., Sukach A., Tkachuk A., Yun I. (2012). Infrared Photodiodes on II–VI and III–V Narrow-Gap Semiconductors. Photodiodes: From Fundamentals to Applications.

[4] Suo F., Tong J., Qian L., Zhang D.H. (2018). Study of dark current in mid-infrared InAsSb-based heteron-i-pphotodiode. J. Phys. D Appl. Phys..

[5] Delli E., Letka V., Hodgson P.D., Repiso E., Hayton J.P., Craig A.P., Lu Q., Beanland R., Krier A., Marshall A.R.J. (2019). Mid-Infrared InAs/InAsSb Superlattice nBn Photodetector Monolithically Integrated onto Silicon. ACS Photonics.

[6] Steenbergen E.H., Morath C.P., Maestas D., Jenkins G.D., Logan J.V. Comparing II–VI and III–V infrared detectors for space applications. Proceedings of the SPIE Defense + Commercial Sensing.

[7] Bouschet M., Zavala-Moran U., Arounassalame V., Alchaar R., Bataillon C., Ribet-Mohamed I., de Anda-Salazar F., Perez J.-P., Péré-Laperne N., Christol P. (2021). Influence of Pixel Etching on Electrical and Electro-Optical Performances of a Ga-Free InAs/InAsSb T2SL Barrier Photodetector for Mid-Wave Infrared Imaging. Photonics.

[8] Wróbel J., Ciupa R., Rogalski A. Performance limits of room-temperature InAsSb photodiodes. Proceedings of the SPIE—The International Society for Optical Engineering.

[9] Schroder D.K., Schroder D.K. (2005). Contact Resistance and Schottky Barriers. Semiconductor Material and Device Characterization.

[10] Mönch W., Kasap S., Capper P. (2017). Electronic Properties of Semiconductor Interfaces. Springer Handbook of Electronic and Photonic Materials.

[11] Schuler-Sandy T., Myers S., Klein B., Gautam N., Ahirwar P., Tian Z.B., Rotter T., Balakrishnan G., Plis E., Krishna S. (2012). Gallium free type II InAs/InAs_x_Sb_1−x_ superlattice photodetectors. Appl. Phys. Lett..

[12] Schuler-Sandy T., Klein B., Casias L., Mathews S., Kadlec C., Tian Z., Plis E., Myers S., Krishna S. (2015). Growth of InAs–InAsSb SLS through the use of digital alloys. J. Cryst. Growth.

[13] Ariyawansa G., Reyner C.J., Duran J.M., Reding J.D., Scheihing J.E., Steenbergen E.H. (2016). Unipolar infrared detectors based on InGaAs/InAsSb ternary superlattices. Appl. Phys. Lett..

[14] Jia B.W., Tan K.H., Loke W.K., Wicaksono S., Lee K.H., Yoon S.F. (2018). Monolithic Integration of InSb Photodetector on Silicon for Mid-Infrared Silicon Photonics. ACS Photonics.

[15] Stareev G., Künzel H., Dortmann G. (1993). A controllable mechanism of forming extremely low-resistance nonalloyed ohmic contacts to group III–V compound semiconductors. J. Appl. Phys..

[16] Lee C.-T., Jaw K.-L., Tsai C.-D. (1998). Thermal stability of Ti/Pt/Au ohmic contacts on InAs/graded InGaAs layers. Solid-State Electron..

[17] Lysczek E.M., Robinson J.A., Mohney S.E. (2006). Ohmic contacts to p-type InAs. Mater. Sci. Eng. B.

[18] Mohney S.E., Lysczek E., Wang S., Robinson J. (2006). Ohmic Contacts to p-Type III–V Semiconductors for the Base of Heterojunction Bipolar Transistors. ECS Trans..

[19] Guo L.W., Lu W., Bennett B.R., Boos J.B., del Alamo J.A. (2015). Ultralow Resistance Ohmic Contacts for p-Channel InGaSb Field-Effect Transistors. IEEE Electron Device Lett..

[20] Goodman P. (2002). Current and future uses of gold in electronics. Gold Bull..

[21] Carraro C., Maboudian R., Magagnin L. (2007). Metallization and nanostructuring of semiconductor surfaces by galvanic displacement processes. Surf. Sci. Rep..

[22] Depestel L.M., Strubbe K. (2004). Electrodeposition of gold from cyanide solutions on different n-GaAs crystal faces. J. Electroanal. Chem..

[23] Topçu E., Dağcı Kıranşan K. (2019). Flexible gold nanoparticles/rGO and thin film/rGO papers: Novel electrocatalysts for hydrogen evolution reaction. J. Chem. Technol. Biotechnol..

[24] Ma Y., Di J., Yan X., Zhao M., Lu Z., Tu Y. (2009). Direct electrodeposition of gold nanoparticles on indium tin oxide surface and its application. Biosens. Bioelectron..

[25] Ye W., Kou H., Liu Q., Yan J., Zhou F., Wang C. (2012). Electrochemical deposition of Au–Pt alloy particles with cauliflower-like microstructures for electrocatalytic methanol oxidation. Int. J. Hydrogen Energy.

[26] Fratini E., Girella A., Saldan I., Milanese C., Dobrovetska O., Sus L., Okhremchuk Y., Kuntyi O., Reshetnyak O. (2015). Nucleation and growth of Au and Au–Pd nanoparticles at the beginning of electrochemical deposition. Mater. Lett..

[27] Sus L., Okhremchuk Y., Saldan I., Kuntyi O., Reshetnyak O., Korniy S. (2015). Controlled gold deposition by pulse electrolysis. Mater. Lett..

[28] González-Buch C., Herraiz-Cardona I., Ortega E.M., Mestre S., Pérez-Herranz V. (2016). Synthesis and characterization of Au-modified macroporous Ni electrocatalysts for alkaline water electrolysis. Int. J. Hydrogen Energy.

[29] Monaico E.V., Monaico E.I., Ursaki V.V., Tiginyanu I.M. (2020). Free-Standing Large-Area Nanoperforated Gold Membranes Fabricated by Hopping Electrodeposition. ECS J. Solid State Sci. Technol..

[30] Vicenzo A., Cavallotti P.L. (2009). Gold electroplating. Gold: Science and Applications.

[31] Grover S., Sahu S., Zhang P., Davis K.O., Kurinec S.K. Standardization of Specific Contact Resistivity Measurements using Transmission Line Model (TLM). Proceedings of the 2020 IEEE 33rd International Conference on Microelectronic Test Structures (ICMTS).

[32] Benyahia D., Kubiszyn Ł., Michalczewski K., Kębłowski A., Martyniuk P., Piotrowski J., Rogalski A. (2016). Molecular beam epitaxial growth and characterization of InAs layers on GaAs (001) substrate. Opt. Quantum Electron..

[33] Wróbel J., Grodecki K., Benyahia D., Murawski K., Michalczewski K., Grzonka J., Boguski J., Gorczyca K., Umana-Membreno G.A., Kubiszyn Ł. Structural and optical characterization of the high quality Be-doped InAs epitaxial layer grown on GaAs substrate. Proceedings of the Thirteenth Integrated Optics: Sensors, Sensing Structures and Methods Conference.

[34] Boguski J., Kolwas K., Kubiszyn Ł., Michalczewski K., Piotrowski J., Wróbel J., Gorczyca K., Kębłowski A., Martyniuk P. (2018). Study on the specific contact resistance of evaporated or electroplated golden contacts to n- and p- type InAs epitaxial layers grown by MBE. Mater. Sci. Semicond. Process..

[35] Kowalewski A., Martyniuk P., Markowska O., Benyahia D., Gawron W. (2016). New wet etching solution molar ratio for processing T2SLs InAs/GaSb nBn MWIR infrared detectors grown on GaSb substrates. Mater. Sci. Semicond. Process..

[36] Kowalewski A., Wróbel J., Boguski J., Gorczyca K., Martyniuk P. (2019). Semiconductor Contact Layer Characterization in a Context of Hall Effect Measurements. Metrol. Meas. Syst..

[37] Beck W.A., Anderson J.R. (1987). Determination of electrical transport properties using a novel magnetic field-dependent Hall technique. J. Appl. Phys..

[38] Antoszewski J., Seymour D.J., Faraone L., Meyer J.R., Hoffman C.A. (1995). Magneto-transport characterization using quantitative mobility-spectrum analysis. J. Electron. Mater..

[39] Dobbelaere W., de Boeck J., van Hove M., Deneffe K., de Raedt W., Mertens R., Borghs G. Long wavelength InA_s02_Sb_0.8_ detectors grown on patterned Si substrates by molecular beam epitaxy. Proceedings of the International Technical Digest on Electron Devices Meeting.

[40] Wu D., Durlin Q., Dehzangi A., Zhang Y., Razeghi M. (2019). High quantum efficiency mid-wavelength infrared type-II InAs/InAs_1−x_Sb_x_ superlattice photodiodes grown by metal-organic chemical vapor deposition. Appl. Phys. Lett..

[41] Stremsdoerfer G., Perrot H., Martin J.R., Cléchet P. (1988). Autocatalytic Deposition of Gold and Palladium onto n-GaAs in Acidic Media. J. Electrochem. Soc..

[42] Jacobs J.W.M., Rikken J.M.G. (1989). Photoelectrochemically-Induced Gold Deposition on p - GaAs Electrodes: Part I. Nucleation and Growth. J. Electrochem. Soc..

[43] Oskam G., Vanmaekelbergh D., Kelly J.J. (1993). The influence of electrodeposited gold on the properties of III–V semiconductor electrodes—Part 1. Results of current—potential measurements on p-GaAs. Electrochim. Acta.

[44] Depestel L.M., Strubbe K. (2003). Influence of the crystal orientation on the electrochemical behaviour of n-GaAs in Au(i)-containing solutions. Phys. Chem. Chem. Phys..

[45] Todeschini M., Bastos da Silva Fanta A., Jensen F., Wagner J.B., Han A. (2017). Influence of Ti and Cr Adhesion Layers on Ultrathin Au Films. ACS Appl. Mater. Interfaces.

[46] Dick K.A., Deppert K., Karlsson L.S., Wallenberg L.R., Samuelson L., Seifert W. (2005). A New Understanding of Au-Assisted Growth of III–V Semiconductor Nanowires. Adv. Funct. Mater..

[47] Jany B.R., Janas A., Piskorz W., Szajna K., Kryshtal A., Cempura G., Indyka P., Kruk A., Czyrska-Filemonowicz A., Krok F. (2020). Towards the understanding of the gold interaction with AIII-BV semiconductors at the atomic level. Nanoscale.

[48] Besikci C., Choi Y.H., Labeyrie G., Bigan E., Razeghi M., Cohen J.B., Carsello J., Dravid V.P. (1994). Detailed analysis of carrier transport in InAs_0.3_Sb_0.7_ layers grown on GaAs substrates by metalorganic chemical-vapor deposition. J. Appl. Phys..

[49] Kim J.D., Wu D., Wojkowski J., Piotrowski J., Xu J., Razeghi M. (1996). Long-wavelength InAsSb photoconductors operated at near room temperatures (200–300 K). Appl. Phys. Lett..

[50] Venter A., Shamba P., Botha L., Botha J.R. (2009). Growth and electrical characterization of Zn-doped InAs and InAs_1−x_Sb_x_. Thin Solid Film..

[51] Kopytko M., Gomolka E., Martyniuk P., Madejczyk P., Rutkowski J., Rogalski A. (2018). High-operating temperature InAsSb/AlSb heterostructure infrared detectors grown on GaAs substrates by molecular beam epitaxy. Opt. Eng..

[52] Chrastina D., Hague J.P., Leadley D.R. (2003). Application of Bryan’s algorithm to the mobility spectrum analysis of semiconductor devices. J. Appl. Phys..

[53] Virtanen P., Gommers R., Oliphant T.E., Haberland M., Reddy T., Cournapeau D., Burovski E., Peterson P., Weckesser W., Bright J. (2020). SciPy 1.0: Fundamental algorithms for scientific computing in Python. Nat. Methods.

